# Acupuncture for HIV-associated distal symmetric peripheral neuropathy: A protocol for systematic review and meta-analysis

**DOI:** 10.1097/MD.0000000000025140

**Published:** 2021-03-12

**Authors:** Ning Gao, Yufeng Guo, Weiming Wang

**Affiliations:** Department of Acupuncture, Guang’ anmen Hospital, China Academy of Chinese Medical Sciences, Beijing, China.

**Keywords:** acupuncture, distal symmetric peripheral neuropathy, human immunodeficiency virus, systematic review

## Abstract

**Background:**

Human immunodeficiency virus (HIV)-associated distal symmetric peripheral neuropathy (DSPN) is one of the most frequent neurological complications of HIV infection, and causes pain and dysaesthesias in millions globally. Many individuals with this infection report using acupuncture to manage their symptoms, but evidence supporting the use of acupuncture is limited. This systematic review will assess the effectiveness and safety of acupuncture for patients with HIV-associated DSPN.

**Methods:**

Databases including MEDLINE, EMBASE, the Cochrane Central Register of Controlled Trials, Scopus, Web of science, AMED (Allied and Complementary Medicine), the Chinese Biomedical Literature Database, the China National Knowledge Infrastructure, Wanfang Database, VIP Database and clinical trials registers (the WHO International Clinical Trials Registry Platform portal and www.ClinicalTrials.gov) will be electronically searched from inception to December 1, 2020. All randomized controlled trials in English or Chinese without restriction on publication status will be included. Selection of studies, extraction of data, and assessment of studies quality will be independently performed by 2 reviewers. The primary outcome measure will be the change in pain intensity assessed by validated scales. Secondary outcomes include change in neurologic summary scores, quality of life, physical function evaluated by admitted tools, and adverse events related to acupuncture reported in the included trials. If possible, a meta-analysis will be conducted to provide an estimate of the pooled treatment effect using Review Manager 5.3 statistical software. Otherwise, qualitative descriptive analysis will be given. The results will be presented as the risk ratio for binary data and the mean difference (MD) or standardized MD for continuous data.

**Results:**

The results of the systematic review will be disseminated via publication in a peer-reviewed journal and presented at a relevant conference.

**Conclusion:**

This review will be the first review entirely focused on assessing the effectiveness and safety of acupuncture for HIV-associated DSPN.

**PROSPERO registration number:**

CRD42020210994.

## 1 Introduction

Distal symmetric peripheral neuropathy (DSPN) is one of the most enfeebled and common complications of human immunodeficiency virus (HIV) infections even with current HIV management strategies.^[1,2]^ The prevalence of DSPN ranges from 9% to 63%^[3]^ among HIV-positive patients, with a higher burden of disease in economically developing countries, especially in Sub-Saharan Africa.^[4]^ The clinical manifestation of HIV-associated DSPN is mainly characterized by paresthesias, symptoms such as burning or tingling pain, numbness in a stocking-glove distribution, ascending symmetrically and predominantly situated in the distal lower extremities.^[5,6]^ These sensorial symptoms, especially stabbing pain, impact patients’ quality of life, generally causing disability in daily routine and loss of productivity, exerting considerable living and economic burden on individuals as well as society.^[7,8]^ According to current research, there are mainly 2 types of HIV-associated DSPN: primary HIV-associated distal symmetric polyneuropathy (HIV-DSP) and antiretroviral therapy toxic neuropathy due to nucleoside reverse transcriptase inhibitors, protease inhibitors, and other toxic antiretroviral medications.^[6,9–12]^ The precise mechanisms of pathogenesis are still ambiguous. While based on several lines of experimental evidence, pathological changes of DSPN can be characterized by local axonal degeneration, with activated macrophages, lymphocytes and glycoprotein 120 infiltrating the dorsal root ganglion, causing local inflammation and maintenance of pain status.^[5]^

Currently, there is still no United States Food and Drug Administration-approved medication for DSPN, and several drugs are used off-label for symptomatic control of neuropathic pain including anticonvulsants, antidepressants, and analgesics.^[13]^ Indeed, the present therapeutic measures for DSPN can only prevent exacerbation and alleviate pain with little efficacy. Also, the potential side effects of the above medicines including insomnia, somnolence, rash, and risk of addiction^[6,13,14]^ may surpass the benefits for HIV-associated DSPN relief. Therefore, novel effective treatments with few side effects need to be explored.

Acupuncture refers to the stimulation of specific acupoints located in the skin and subcutaneous tissues by manual insertion of needles.^[15]^ As an indispensable therapy of traditional Chinese medicine, acupuncture has been extensively used over recent decades around the globe.^[16]^ Many studies have suggested acupuncture has an analgesic effect on lower limb pain^[17,18]^ or hyperalgesia. According to current research, potential mechanisms about acupuncture reducing central sensitisation and improving hyperalgesia involve segmental inhibition, the release of endogenous opioid peptides, adrenergic and serotonin, as well as the reduction in the levels of inflammatory mediators.^[18]^ During the previous years, 2 systematic reviews focused on evaluating the efficacy and safety of acupuncture for general peripheral neuropathic pain conditions from various etiologies.^[19,20]^ Another 2 studies respectively assessed the effect of physical therapy and non-pharmacologic treatments^[21,22]^ for HIV-associated neuropathy pain. However, these studies vary considerably in terms of study types or therapeutic methods, and are not entirely specific to acupuncture regimen for HIV-associated DSPN. The overall conclusions were unclear^[19]^ or indicate that acupuncture has a marginal beneficial effect^[20]^ on HIV-associated neuropathy pain. Thus, the clinical evidence for HIV-associated DSPN is still inadequate.

This article presents a protocol for assessing the evidence on the effectiveness and safety of acupuncture treatment for HIV-associated DSPN based on the most comprehensive and up-to-date resources.

## 2 Methods

This protocol was drafted in accordance with the Preferred Reporting Items for Systematic reviews and Meta-Analysis Protocols.

### 2.1 Criteria for considering studies for this review

#### 2.1.1 Types of studies

All randomized controlled trials (RCTs) of acupuncture therapy for HIV-associated DSPN will be included. There are no restrictions on publication status. The language is limited to Chinese and English. Uncontrolled clinical trials, quasi-RCTs, and animal studies will be excluded. If there is reanalysis literature based on prior RCTs, we will only extract data derived from original studies.

#### 2.1.2 Types of participants

Participants with a definite diagnosis of HIV-associated DSPN based on at least one of the current or past definitions or guidelines for HIV-associated DSPN will be included, regardless of their sex, age, race, or background.

#### 2.1.3 Types of interventions

Acupuncture therapy is defined as the stimulation of acupuncture points by any method including electroacupuncture, manual acupuncture, body acupuncture, scalp acupuncture, ear acupuncture, fire needling, elongated needle, intradermal needling, percussopunctator, dry needling, and laser acupuncture. The control intervention could be placebo acupuncture, sham acupuncture, no treatment (waiting list control), routine treatment, or other treatments. The review will draw the following comparisons: any type of acupuncture compared with placebo acupuncture, sham acupuncture, no treatment (waiting list control), routine treatment, or other treatment; any type of acupuncture in combination with another therapy compared with the other treatment alone. Trials comparing different types of acupuncture therapy will be excluded.

#### 2.1.4 Types of outcome measures

##### 2.1.4.1 Primary outcomes

Changes in pain intensity reported by participants using validated scales (e.g., visual analogue scales, numerical rating scales, Gracely Pain Scale).

##### 2.1.4.2 Secondary outcomes

1. Change in neurologic summary scores assessed by validated instruments.
2. Change in quality of life measured by any validated instruments.
3. Change in physical function evaluated by admitted tools.
4. Any adverse events related to acupuncture as reported in the included trials.

### 2.2 Search methods for identification of studies

#### 2.2.1 Electronic searches

The electronic search strategies will be designed to search relevant references in the following databases and clinical trials registry, including MEDLINE, EMBASE, the Cochrane Central Register of Controlled Trials, Scopus, Web of science, AMED (Allied and Complementary Medicine), the Chinese Biomedical Literature Database, the China National Knowledge Infrastructure, Wanfang Database, VIP Database, the WHO International Clinical Trials Registry Platform portal and www.ClinicalTrials.gov. An electronic search will be done from inception to December 1, 2020. All RCTs in English or Chinese without restriction on publication status will be included. The search will be performed in English and Chinese. The following terms will be used: acquired immune deficiency syndrome, acquired immune deficiency syndrome, HIV, human immunodeficiency virus, HIV-related neuropathic pain, HIV-related distal symmetric polyneuropathy, HIV-related distal sensory peripheral neuropathy, HIV-associated distal sensory polyneuropathy, HIV-associated painful peripheral neuropathy, painful HIV-associated sensory neuropathy, HIV-PPN, HIV-DSP, HIV-DSPN; acupuncture, body acupuncture, scalp acupuncture, manual acupuncture, auricular acupuncture, ear acupuncture, electroacupuncture, fire needling, dermal needle, plum blossom needle, abdominal acupuncture, filiform steel needle (Table 1 details the search strategy for Embase).

**Table 1:** Searching strategy for EMBASE.

Database	Search strategy
EMBASE Search: 19	1 Acquired immune deficiency syndrome.mp. or exp acquired immune deficiency syndrome/(143039) 2 Human immunodeficiency virus.mp. or exp Human immunodeficiency virus/(451436) 3 HIV infection.mp. or exp Human immunodeficiency virus infection/(389532) 4 (“HIV disease” or “HIV-1” or “AIDS” or “HIV”).mp. [mp=title, abstract, heading word, drug trade name, original title, device manufacturer, drug manufacturer, device trade name, keyword, floating subheading word, candidate term word] (504177) 5 1 or 2 or 3 or 4 (606231) 6 Peripheral neuropathy.mp. or exp peripheral neuropathy/(78236) 7 exp neuropathy/ or neuropathy.mp. (776898) 8 neuropathic pain.mp. or exp neuropathic pain/(42689) 9 exp polyneuropathy/ or Polyneuropathy.mp.(46515) 10 Peripheral nervous system.mp. or exp peripheral nervous system/(388239) 11 exp neuralgia/ or Neuralgia.mp.(108374) 12 (“Neuropathic”or “Distal sensory polyneuropathy” or “Distal sensory peripheral neuropathy” or “Polyneuropathies” or “Peripheral nervous system diseases”).mp. [mp=title, abstract, heading word, drug trade name, original title, device manufacturer, drug manufacturer, device trade name, keyword, floating subheading word, candidate term word] (57965) 13 6 or 7 or 8 or 9 or 10 or 11 or 12 (1050779) 14 5 and 13 (15385) 15 (“HIV-related neuropathy” or “HIV-associated neuropathy” or “HIV-related neuropathic pain” or “HIV-related distal symmetric polyneuropathy” or “HIV-related distal sensory peripheral neuropathy” or “HIV-associated distal sensory polyneuropathy” or “HIV-associated painful peripheral neuropathy” or “Painful HIV-associated sensory neuropathy” or “HIV-PPN” or “HIV-DSP” or “HIV-DSPN”).mp. [mp=title, abstract, heading word, drug trade name, original title, device manufacturer, drug manufacturer, device trade name, keyword, floating subheading word, candidate term word] (206) 16 14 or 15 (15385) 17 Acupuncture.mp. or exp acupuncture/(49939) 18 auricular acupuncture.mp. or exp auricular acupuncture/(817) 19 electroacupuncture.mp. or exp electroacupuncture/(7760) 20 (“acupuncture therapy” or “body acupuncture” or “scalp acupuncture” or “manual acupuncture” or “auricular acupuncture” or “ear acupuncture” or “electroacupuncture” or “fire needling” or “dermal needle” or “plum blossom needle” or “abdominal acupuncture” or “filiform steel needle”).mp. [mp=title, abstract, heading word, drug trade name, original title, device manufacturer, drug manufacturer, device trade name, keyword, floating subheading word, candidate term word](10736) 21 17 or 18 or 19 or 20 (50039) 22 placebo$.tw. (313648) 23 random$.tw. (1578182) 24 factorial$.tw. (39024) 25 crossover$.tw. (77304) 26 cross-over$.tw.(33070) 27 cross over$.tw. (33070) 28 (doubl$ adj blind$).tw. (213004) 29 (singl$ adj blind$).tw. (25652) 30 assign$.tw.(403147) 31 allocat$.tw.(157455) 32 volunteer$.tw.(260847) 33 Crossover Procedure/(64550) 34 double-blind procedure.tw.(239) 35 Randomised Controlled Trial/(621922) 36 Single Blind Procedure/(40312) 37 22 or 23 or 24 or 25 or 26 or 27 or 28 or 29 or 30 or 31 or 32 or 33 or 34 or 35 or 36 (2374339) 38 16 and 21 and 37 (19) 39 limit 38 to human (19)

#### 2.2.2 Searching other resources

We will also search the reference lists of included trials, relevantly published reviews, and Google Scholar for potential qualifying studies.

### 2.3 Data collection and analysis

#### 2.3.1 Selection of studies

**Figure 1:**
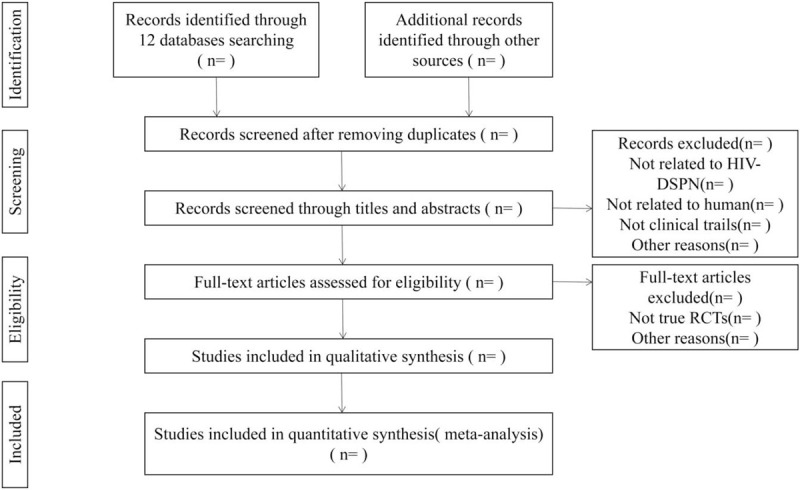
Flow diagram of the study selection process.

Two reviewers (Ning Gao and Yufeng Guo) will individually screen titles and abstracts of all literature identified. The full text of publications possibly related to the review will be downloaded to assess relevance based on the predefined inclusion/exclusion criteria. The final decision will be arbitrated by Weiming Wang and resolved by consensus, if there are any discrepancies. The whole process of study selection and drop reasons will be illustrated in a Preferred Reporting Items for Systematic reviews and Meta-Analyses (PRISMA) flow chart (Fig. 1).

#### 2.3.2 Data extraction and management

Two reviewers (Ning Gao and Yufeng Guo) will separately extract data from the included studies using a predesigned data extraction table. The following information will be abstracted: identification features, participants (e.g., sample size, age range, gender distribution, diagnostic criteria, disease course), intervention (e.g., type of acupuncture therapy, study duration, and follow-up duration), comparison (e.g., no treatment, placebo therapy or other active treatment), outcomes (e.g., type of outcome measures, reported outcomes, adverse events), study design (e.g., randomization, inclusion/ exclusion criteria, and withdrawals/dropouts). Abstracted data will be proofread mutually by 2 reviewers for completeness and accuracy and double-checked by a third reviewer if necessary. Different opinions will be solved after the consultation with Weiming Wang and reach an agreement before imported into Review Manager 5.3.

#### 2.3.3 Assessment of risk of bias in included studies

Two reviewers (Ning Gao and Yufeng Guo) will separately evaluate the risk of bias for the included studies according to the criteria sketched in the Cochrane Collaboration's tool, which will involve the following aspects:

1. Selection bias: random sequence generation and allocation concealment;
2. Performance bias: blinding of investigators, participants, and care providers;
3. Detection bias: blinding of outcome assessors;
4. Attrition bias: incomplete outcome data;
5. Reporting bias: selective reporting; and
6. Other bias such as conflicts of interest.

The following grades will present management of the risk of bias for each aspect: “low risk,” “high risk,” or “unclear.” The quality of included trials will be evaluated based on the Grading of Recommendations Assessment, Development and Evaluation instrument, ranked in 4 grades: high, moderate, low, and very low. Any discrepancies will be resolved by consultation with Weiming Wang.

#### 2.3.4 Measures of treatment effect

Effect size from included studies will be synthesized and analyzed by RevMan V.5.3. Supposing that the continuous outcome variables were measured by different scales, the standardized mean difference will be used with 95% confidence intervals (CIs) to measure the treatment effect. Otherwise, the mean difference (MD) will be used with 95% CIs. Regarding the dichotomous data, we will analyze treatment effects through a risk ratio with 95% CIs. Other binary data will be transformed into the risk ratio risk ratio modality.

#### 2.3.5 Unit of analysis issues

The unit of analysis will be each patient in the included trials. For studies that have more than 2 treatment arms or special types of study design, we plan to handle these in accordance with guidance provided in the Cochrane Handbook for Systematic Reviews of Interventions.^[23]^

#### 2.3.6 Dealing with missing data

For every included study, the quantities of withdrawals, exclusions parts, and missing data will be gathered and request the corresponding author for incomplete information via E-mail or telephones. If we fail to achieve it, we will assume dichotomous missing outcomes for participants not obtaining any improvement in their clinical outcome variables. For continuous missing outcomes, we will attempt to calculate mean deviation and standard deviation values newly as the first option when the medians, *P* values or CIs are recorded in the included studies. When necessary, the possible impact of missing data on the final findings of the review will be documented in the discussion section.

#### 2.3.7 Assessment of heterogeneity

We will carry out the Cochrane's Q test for the detection of heterogeneity. The *I*^2^ statistic will be calculated to measure the impact of heterogeneity among the trials. An *I*^2^ value greater than or equal to 50% suggests a substantial level of inconsistency. If we identify substantial heterogeneity, we will document it and detect potential causes by conducting prespecified subgroup analysis.

#### 2.3.8 Assessment of reporting bias

If included studies exceed 10, we will construct a funnel plot and Egger test to explore possible small study bias. We will also provide a careful interpretation of the results based on several explanations for the funnel plot's asymmetry.

#### 2.3.9 Data synthesis

In case of 2 or more eligible RCTs are included, meta-analysis will be conducted with Review Manager 5.3. The outcomes of the χ^2^ test and *I*^2^ test for heterogeneity determine whether fixed-effects model or random-effects model will be used. If substantial heterogeneity is found (*I*^2^ value no less than 50%), we will use random-effects model as the first option; otherwise (*I*^2^ value less than 50%), we will adopt fixed-effects model. If considerable heterogeneity exists in the trials, subgroup analyses will be conducted attempting to detect the source of heterogeneity. If we fail to explore the source of considerable heterogeneity, a narrative qualitative summary will substitute for meta-analysis providing characteristics and findings of the included studies systematically.

#### 2.3.10 Subgroup analysis and investigation of heterogeneity

We plan to conduct subgroup analyses for comparing races (Asians or Europeans or Africans) and sex of included participants to explore potential heterogeneity of the results, if possible.

#### 2.3.11 Sensitivity analysis

We will perform sensitivity analysis to confirm the firmness of the conclusion in this review on the basis of different levels of bias of the included RCTs. In the following analysis, lower quality studies will be removed, then we will compare the results with those adopting the worst-case strategy to merge trials. A discussion will be carried out to determine if the lower quality RCTs should be excluded, concerning their sample size, levels of evidence and influence on pooled effect size.

### 2.4 Ethics and dissemination

Formal ethical approval is not required because the existing data will be not personalized. The results of the systematic review will be disseminated via publication in a peer-reviewed journal and presented at a relevant conference.

## 3 Discussion

A great pain burden exists in acquired immune deficiency syndrome patients with DSPN with increased life expectancies,^[24]^ cohort studies indicate levels of depression were positively correlated with pain intensity and reduced quality of life,^[25]^ and it is hard to treat via pharmacology in particular.^[26,27]^ Although several RCTs of acupuncture for HIV-associated DSPN have been reported to date, the evidence for its effectiveness and adverse events has not been specifically evaluated. This study will synthesize the current evidence on acupuncture for patients with HIV-associated DSPN. The results will help provide high-quality evidence on the effectiveness and safety of acupuncture for HIV-associated DSPN, thus enabling patients, acupuncturists, and health policymakers to make the proper choice regarding treatment for HIV-associated DSPN.

This review will be the first review entirely specific to assessing the effectiveness and safety of acupuncture for HIV-associated DSPN. However, some potential limitations still exist. First, there may be heterogeneity among the included studies due to the use of different evaluation criteria as well as acupuncture techniques. Then, an incomplete coverage of current studies possibly occur, as certain relevant literatures may lack predefined outcomes indicators or still be hard to retrieve with comprehensive search strategies. Another likely issue is relevant to quality of the evidence due to methodological reporting in certain published articles, which may engender partial low quality of the evidence. We will consider it in the process of sensitivity analyses in terms of methodological quality and document relevant results in the study.

## Author contributions

Weiming Wang contributed to study conception. Ning Gao drafted the protocol and then it was revised by Weiming Wang. Ning Gao and Yufeng Guo will independently screen the potential studies, and independently assess the risk of bias of the included studies. Weiming Wang will arbitrate any disagreements during the review. Ning Gao and Yufeng Guo will extract data. Weiming Wang will perform data synthesis. All authors read and approved the final verison of the manuscript.

**Conceptualization:** Weiming Wang.

**Writing – original draft:** Ning Gao, Yufeng Guo.

**Writing – review & editing:** Weiming Wang.
